# X-Band Parallel-Mode and Multifrequency Electron Paramagnetic Resonance Spectroscopy of *S* = 1/2 Bismuth Centers

**DOI:** 10.1021/acs.inorgchem.2c01141

**Published:** 2022-07-14

**Authors:** Julia Haak, Julia Krüger, Nikolay V. Abrosimov, Christoph Helling, Stephan Schulz, George E. Cutsail III

**Affiliations:** † Max Planck Institute for Chemical Energy Conversion (CEC), Stiftstraße 34−36, 45470 Mülheim an der Ruhr, Germany; ‡ Institute of Inorganic Chemistry, University of Duisburg-Essen, Universitätsstraße 5-7, 45141 Essen, Germany; § Center for Nanointegration Duisburg-Essen (CENIDE), University of Duisburg-Essen, Universitätsstraße 5-7, 45141 Essen, Germany; ∥ Leibniz-Institut für Kristallzüchtung, Max-Born Strasse 2, 12489 Berlin, Germany

## Abstract

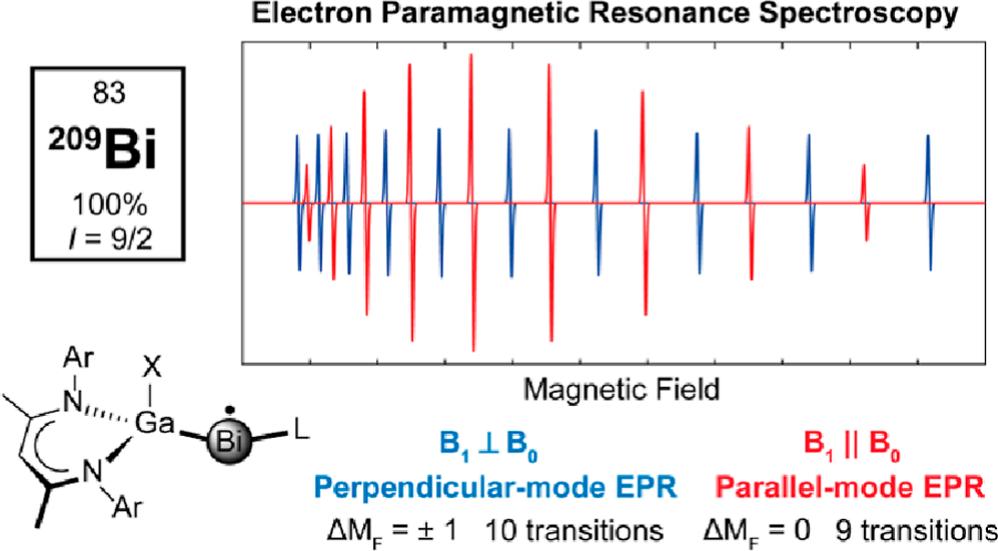

The recent successes in the isolation and characterization of several bismuth radicals inspire the development of new spectroscopic approaches for the in-depth analysis of their electronic structure. Electron paramagnetic resonance (EPR) spectroscopy is a powerful tool for the characterization of main group radicals. However, the large electron–nuclear hyperfine interactions of Bi (^209^Bi, *I* = 9/2) have presented difficult challenges to fully interpret the spectral properties for some of these radicals. Parallel-mode EPR (*B*_1_∥*B*_0_) is almost exclusively employed for the study of *S* > 1/2 systems but becomes feasible for *S* = 1/2 systems with large hyperfine couplings, offering a distinct EPR spectroscopic approach. Herein, we demonstrate the application of conventional X-band parallel-mode EPR for *S* = 1/2, *I* = 9/2 spin systems: Bi-doped crystalline silicon (Si:Bi) and the molecular Bi radicals [L(X)Ga]_2_Bi^•^ (X = Cl or I) and [L(Cl)GaBi(^Me^cAAC)]^•+^ (L = HC[MeCN(2,6-*i*Pr_2_C_6_H_3_)]_2_). In combination with multifrequency perpendicular-mode EPR (X-, Q-, and W-band frequencies), we were able to fully refine both the anisotropic *g*- and *A*-tensors of these molecular radicals. The parallel-mode EPR experiments demonstrated and discussed here have the potential to enable the characterization of other *S* = 1/2 systems with large hyperfine couplings, which is often challenging by conventional perpendicular-mode EPR techniques. Considerations pertaining to the choice of microwave frequency are discussed for relevant spin-systems.

## Abstract 2

The extremely large electron−nuclear hyperfine interaction of bismuth enables the application of parallel-mode electron paramagnetic resonance (EPR) spectroscopy for these *S* = 1/2, *I* = 9/2 spin systems, as demonstrated for Bi-doped Si (Si:Bi) and several bismuth-centered molecular radicals. Combining this approach with multifrequency perpendicular-mode EPR spectroscopy (X-, Q-, and W-band frequencies) successfully determines the EPR parameters of these challenging Bi radicals.

## Introduction

The recent isolations and characterizations of stable bismuth radicals are part of an advancing and curious field in heavy main group chemistry due to their potential applications in synthesis and catalysis.^1−4^ The identification of bismuth radicals as catalytic intermediates for different coupling reactions necessitates the spectroscopic identification and detailed characterization of these centers.^5,6^ Given that only a handful of stable molecular bismuth radicals have been isolated and characterized thus far,^7−11^ general spectroscopic properties and trends within these are yet to be determined. In each case, unique chemical, electronic, or magnetic properties of the bismuth radical influence or dictate what spectroscopic approaches are best suited for their characterization.

**Scheme 1:**
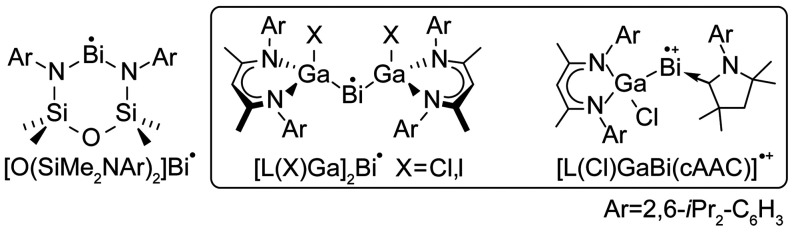
Molecular Bismuth Radicals [O(SiMe_2_NAr)_2_]Bi^•^,^10^ [L(X)Ga]_2_Bi^•^,^11,14^ and [L(Cl)GaBi(^Me^cAAC)]^•+^.^13^

Due to their paramagnetic nature, electron paramagnetic resonance (EPR) spectroscopy plays a central role in the characterization of bismuth radicals.^12^ For bismuth, a single nuclear-active isotope, ^209^Bi, occurs at 100% natural abundance with a nuclear spin of *I* = 9/2. To date, very few examples of stable bismuth radicals exist, and the complete electron paramagnetic characterization of their hyperfine interaction is often challenging. The first stable molecular bismuth radical to be fully characterized by multifrequency EPR was [O(SiMe_2_NAr)_2_]Bi^•^ (Ar = 2,6-*i*Pr_2_C_6_H_3_),^10^ which was found to have a very large isotropic hyperfine coupling (|*a*_iso_| ∼ 3800 MHz), among the largest hyperfine interactions measured yet by EPR spectroscopy.^10^ Previously, we have demonstrated the *S* = 1/2 radical behavior of a homoleptically gallium-coordinated bismuth radical, [L(I)Ga]_2_Bi^•^ (L = HC[C(Me)N(2,6-*i*Pr_2_C_6_H_3_)]_2_), Scheme 1.^11^ Variable-temperature magnetic susceptibility measurements yielded a response consistent with *S* = 1/2 and *g*_av_ = 2.05. The ∼4 K perpendicular-mode X-band (∼9.6 GHz) EPR spectrum of this radical exhibited numerous broad features from 0 to >7000 G. The higher frequency Q-band (∼34 GHz) echo-detected EPR failed to resolve distinct *g*-values or components of the anisotropic hyperfine tensor. More recently, we reported the general synthesis and characterization of heteroleptically coordinated group 15 radicals, stabilized by L(X)Ga and a cyclic (alkyl)(amino)carbene (^Me^cAAC) ligand (^Me^cAAC = [H_2_C(CMe_2_)_2_N(2,6-*i*-Pr_2_C_6_H_3_)]C). Similar to [L(I)Ga]_2_Bi^•^, [L(Cl)GaBi(^Me^cAAC)]^•+^ exhibited a broad perpendicular-mode X-band EPR of numerous transitions.^13^ Unfortunately, the determination of accurate EPR parameters from these previous EPR experiments was not feasible, inspiring the development of additional spectroscopic methods and techniques for the characterization of these unique and complex radicals.

The EPR response of the *S* = 1/2 bismuth radical may be approximated with the classic spin Hamiltonian, , where the first term is the electronic Zeeman interaction and the second term is the electron–nuclear hyperfine interaction. For the Zeeman interaction, μ_B_ is the Bohr magneton, **B** is the external magnetic field, and **g** is the electronic **g** tensor. For the hyperfine interaction, **A** is the hyperfine tensor.

The most commonly employed mode of EPR spectroscopy is perpendicular-mode EPR spectroscopy, where the microwave field (*B*_1_) is perpendicular to the magnetic field (*B*_0_): *B*_1_ ⊥ *B*_0_. In the high-magnetic field regime, the Zeeman interaction is much larger than the hyperfine interaction, allowing for the hyperfine interaction to be treated as a perturbation of the Zeeman interaction. In this regime, the possible spin-states may be described by their quantum numbers |*M*_S_, *M*_I_⟩, and transitions that obey the classic selection rules of Δ*M*_S_ = 1, Δ*M*_I_ = 0 are observed. This is in contrast to the low-field limit where the Zeeman and the hyperfine interactions cannot be treated sequentially, and the magnitude of these interactions can be comparable.

For an isotropic *S* = 1/2, *I* = 9/2 spin system, one would observe 10 transitions in the perpendicular-mode EPR experiment. In fact, this is observed for Bi-doped crystalline silicon (Si:Bi) recorded at the X-band frequency.^15−17^ This material is of interest due to its large number of transition states and potential applications in quantum computing.^16−20^ The Si:Bi system is well characterized with isotropic *g* = 2.0047 and an isotropic Bi hyperfine coupling of *a*_iso_ = 1475.2 MHz. We do note that the 10-line pattern observed in Si:Bi at X-band frequencies is not equally split as it is in what we will simply refer to as the moderate-field regime. Here, the Zeeman and the hyperfine interactions are of comparable magnitude with the maximum number of transitions between the possible states being observed. At frequencies lower than the X-band, and into the low-field regime, the number of observable transitions may be reduced, which will be discussed in more detail later. Simply going to higher microwave frequencies, where larger magnetic field strengths are required to observe the resonances, the 10-line pattern will become evenly spaced as the Zeeman interaction becomes significantly greater than the hyperfine interaction.

Parallel-mode EPR, *B*_1_∥*B*_0_, is possible on common X-band EPR spectrometers with commercially available or home-built microwave cavities^21^ (or resonators), where some are “dual-mode”, allowing the spectroscopists to easily select either the perpendicular- or parallel-mode. Historically, parallel-mode EPR spectroscopy has typically been used to explore *S* > 1/2 systems^22−26^ where the zero-field splitting permits admixing of electronic spin-states and observed transitions obey a Δ*M*_S_ ≠ ± 1 selection rule (i.e., Δ*M*_S_ = 0 or Δ*M*_S_ = 2). For the study of *S* = 1/2 centers, parallel-mode EPR has only found very limited applications (if any) as a majority of *S* = 1/2 spin systems in the high-magnetic field regime have no transitions fulfilling the classic ‘Δ*M*_S_ ≠ ± 1’ selection rule.

As alluded to earlier, systems with large hyperfine interactions, such as Si:Bi, may not be in the high-field regime at conventional microwave frequencies but in a so-called “moderate-field regime”, where Zeeman and hyperfine interactions are closer in magnitude and all allowed transitions are observed, or even in the low-field regime, where the Zeeman interaction is much smaller than the hyperfine interaction. For both of these cases, off-diagonal terms cannot be neglected anymore, and the classic EPR selection rules need to be reconsidered. As previously derived for the case of the hydrogen atom by Weil,^27,28^*M*_S_ and *M*_I_ are not necessarily good quantum numbers in the low- and moderate-field regimes, but rather spin-states should be noted as |*F*, *M*_F_⟩, the eigenstates of *F*^2^ and *F*_*z*_ of the total angular momentum: **F** = **S** + **I**. From this, one may simply rederive the selection rules for both perpendicular and parallel modes as Δ*M*_F_ = ±1 and Δ*M*_F_ = 0, respectively. The Δ*M*_F_ = 0 selection rule has curious implications because for *S* = 1/2 systems with large hyperfine coupling at low or moderate fields, this predicts allowed parallel-mode EPR transitions.

Weil initially predicted the allowed transitions for the *S* = 1/2, *I* = 1/2 hydrogen atom (*a*_iso_ = 1420 MHz), along with their intensities.^27^ Later, Mitrikas et al.^29^ directly measured both the perpendicular- and parallel-mode EPR spectra of the H atom encapsulated in polyhedral oligomeric silsesquioxane cages (^1^H@*h*_72_Q_8_M_8_) and observed a hyperfine coupling of *A* = 1416.58 MHz, in excellent agreement with Weil’s proposal. The parallel-mode EPR of the H atom is fairly weak, and it is noted that the intensity of both the perpendicular- and parallel-mode EPR transitions is determined by the time-dependent perturbation solution of the magnetic dipole transition spin-Hamiltonian operator. Therefore, their intensities have only a magnetic field dependence.^27^ Weil proposed that at 2 GHz, the H atom is completely in the low-field regime, and the intensities of both the parallel- and perpendicular-mode EPR spectra would be comparable, Figure S1. More simply stated, the intensity of the parallel-mode EPR is proportional to the degree of state mixing, which is greatest in the low-field regime. Mitrikas et al.^29^ recognized the potential of parallel-mode EPR spectroscopy compared to other *S* = 1/2 systems and reported the predicted perpendicular- and parallel-mode EPR spectra of Si:Bi. Recently, the extremely large hyperfine interaction of *a*_iso_ = 3467 MHz in a *S* = 1/2 Lu(II) complex allowed the observation of parallel-mode transitions in perpendicular-mode X-band EPR spectra via strong overcoupling of the resonator to maximize the bandwidth, allowing mixed detection of parallel- and perpendicular-mode EPR transitions.^30^ While the pure parallel-mode EPR spectrum was not reported, the study is a rare example of parallel-mode EPR for *S* = 1/2 systems with large hyperfine couplings, encouraging its application for bismuth centers.

We first further explored the experimental feasibility of parallel-mode X-band EPR applied to *S* = 1/2 systems through the study of Si:Bi. The previous challenges we faced characterizing molecular Bi radicals, our successful multifrequency EPR approaches to other group 15 radicals, and the prospect of parallel-mode EPR to yield even further insights inspired us to continue our spectroscopic studies of [L(I)Ga]_2_Bi^•^ and [L(Cl)GaBi(^Me^cAAC)]^•+^. Moreover, the recently isolated [L(Cl)Ga]_2_Bi^•^ radical featuring Cl substituents instead of the I substituents of the structurally analogous [L(I)Ga]_2_Bi^•^ was included in this study.^14^ Together, the parallel-mode and multifrequency perpendicular-mode EPR spectra offer the most complete characterization of these radicals thus far. Lastly, the application of parallel-mode EPR spectroscopy to *S* = 1/2 spin systems has rarely been demonstrated thus far, and we will further illustrate the capabilities of conventional EPR spectroscopies, including parallel-mode EPR, to these emerging Bi radical systems and discuss more broadly its application to systems with large hyperfine interactions.

## Results and Discussion

**Figure 1:**
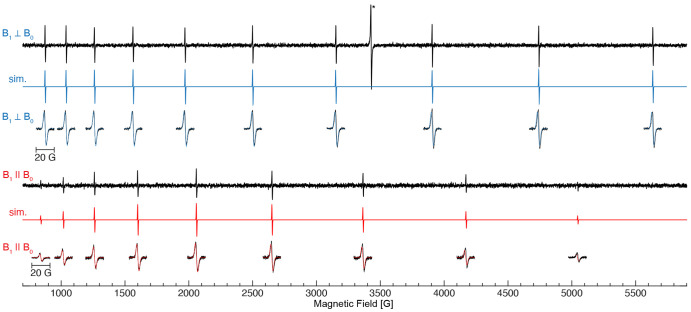
Wide magnetic field X-band perpendicular- (∼9.65 GHz) and parallel-mode (∼9.37 GHz) EPR scans of Si:Bi taken at 25 K. Simulations for each mode are given in color, calculated from the same spin parameters of *g* = 2.0047 and *A*(^209^Bi) = 1475.5 MHz and a 3.7 G (peak-to-peak) linewidth. Narrow magnetic field (20 G) scans are centered at each transition field position. Spectrometer conditions are described in Materials and Methods. The asterisk (*) denotes a *g* = 2.0097 radical impurity.

The X-band (∼9.65 GHz) perpendicular-mode EPR spectrum of Si:Bi exhibited an intense unsaturated signal around 25 K. Attempts to collect EPR spectra at lower temperatures gave spectra that were easily saturated, consistent with previous observations.^16^ Warming of the sample to temperatures above 40 K yielded weaker signals as expected prior to complete spoiling of the tuning due to a rapid decrease of the observed *Q*-factor of the cavity. The X-band perpendicular-mode EPR spectrum exhibits 10 transitions following the typical 2*I* + 1 hyperfine splitting pattern. The transitions are of approximately equal intensity but inequivalent magnetic field spacing. A sharp, more intense transition is observed at 3429 G, *g* = 2.0097 and is attributed to nonspecific radicals in the Teflon material holding the Si:Bi crystal. The EPR spectrum of the Si:Bi crystal is in excellent agreement with that previously published at X-band frequencies. The Si:Bi perpendicular-mode EPR spectrum exhibits very narrow Gaussian line shapes that are 3.7 G peak-to-peak, Figure 1. The spectrum is well reproduced by simulations using an isotropic *g* = 2.0047 and *A*(^209^Bi) = 1475.5 MHz, in agreement with the EPR parameters previously reported.^15−17^

**Figure 2:**
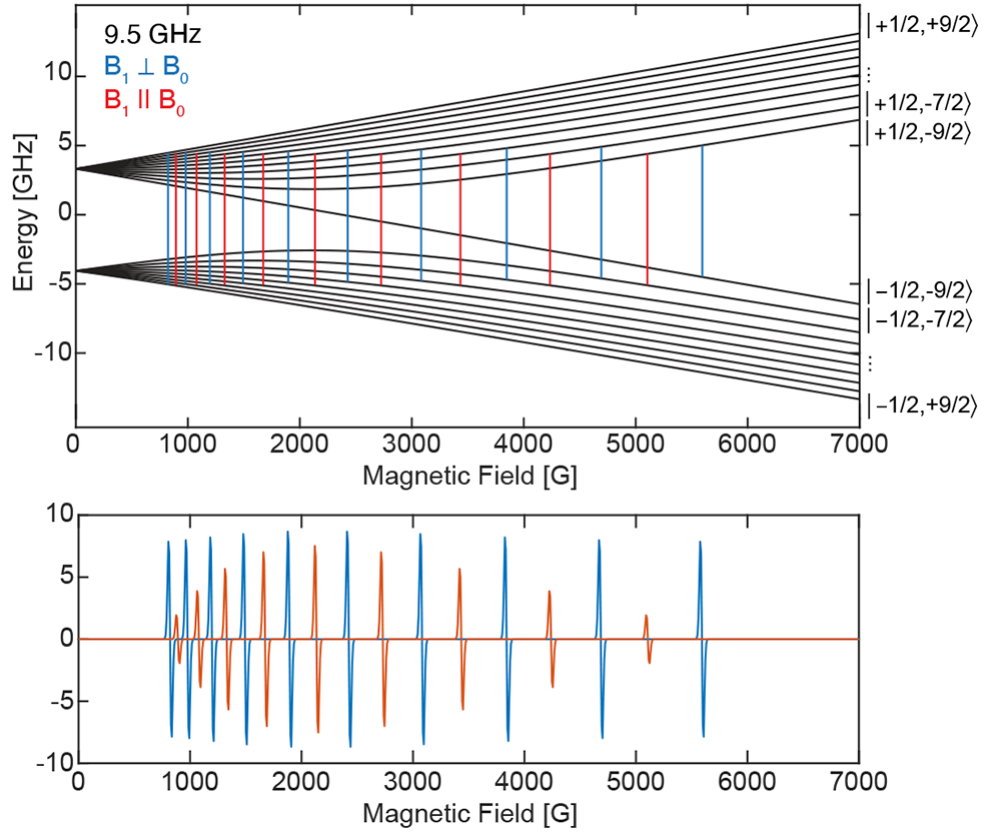
Energy level (Breit–Rabi) diagram of Si:Bi with predicted transitions for parallel and perpendicular modes and the resulting simulated EPR spectrum for a microwave frequency of 9.5 GHz. The transitions are calculated from the isotropic *g* = 2.0047 and *A*(^209^Bi) = 1475.5 MHz. Despite *M*_S_ and *M*_I_ not being good quantum numbers in the low-field experiment, the individual levels are labeled with the notation |*M*_S_, *M*_I_⟩ for ease of reading.

The parallel-mode X-band (∼9.37 GHz) EPR spectrum was also recorded at 25 K, Figure 1. The spectrum exhibits a clear nine-line pattern, one less transition than the perpendicular-mode EPR spectrum. The number of predicted transitions is the result of the Δ*M*_F_ = 0 selection rule for parallel-mode EPR, yielding a 2*I* hyperfine splitting pattern, Figure 2. Furthermore, the parallel-mode EPR spectrum exhibits inequivalent transition intensities, with the maximum intensity at the fifth and the sixth transitions. Similar to the perpendicular-mode EPR spectrum, the parallel-mode EPR spectrum exhibits transitions that are 3.7 G wide (peak-to-peak). It is important to note that the radical impurity signal observed at *g* = 2 in the perpendicular-mode EPR spectrum is completely absent in the parallel-mode spectrum as there are no allowed transitions for *S* = 1/2 with no/weak hyperfine couplings. Simulations of the parallel-mode faithfully reproduce both the transition positions and their intensity patterns, Figure 1.

For the Si:Bi system, the EPR spectrum is collected in the moderate-field regime as the hyperfine features observed in both EPR detection modes are inequivalently spaced. The parallel-mode EPR spectrum of Si:Bi exhibits a less intense spectrum compared to the perpendicular-mode EPR spectrum. Weil previously described the parallel-mode EPR transition intensity mechanism of the hydrogen atom and showed that the intensity of the parallel-mode transitions decreases as one leaves the low-field regime and moves toward the high-field regime, and the degree of spin-state mixing decreases. Simulations of the Si:Bi parallel-mode EPR response intensity as a function of microwave frequency predict a constant decrease in intensity away from the maximum response at approximately 7.5 GHz, Figure S2. Therefore, the decreased intensity of the parallel-mode spectrum, due to decreased state mixing in the moderate-field regime at ∼9.5 GHz, is a foreseeable phenomenon. One would predict approximately equally intense perpendicular- and parallel-mode EPR spectra at field strengths required for an ∼8 GHz EPR experiment. These results immediately demonstrate the ability and serve as a proof-of-concept that parallel-mode EPR spectroscopy of an *S* = 1/2, *I* = 9/2 system with large hyperfine interactions is not only feasible but is easily obtained.

**Figure 3:**
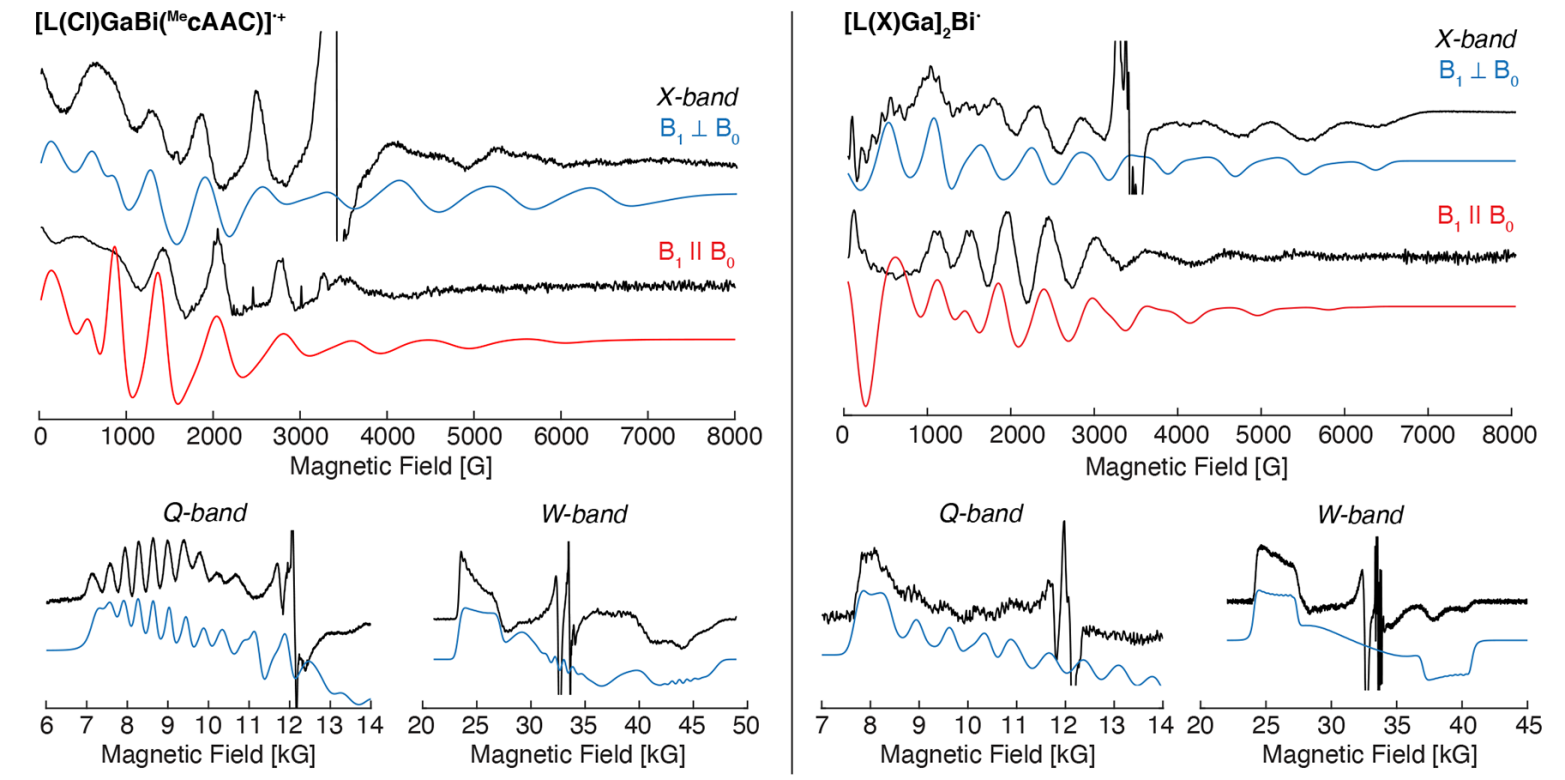
X-band perpendicular- (B_1_⊥B_0_, ∼9.63 GHz) and parallel-mode (B_1_∥B_0_, ∼9.33 GHz) EPR spectra in addition to the numerical derivative of two-pulse-detected Q-band (∼34.0 GHz) and W-band (∼94.0 GHz) EPR spectra of [L(Cl)GaBi(^Me^cAAC)]^•+^ (left) and [L(X)Ga]_2_Bi^•^ (right). Simulations for each EPR experiment are given in color, calculated from the same spin parameters for each molecule. [L(Cl)GaBi(^Me^cAAC)]^•+^: **g** = [2.67, 1.95, 1.54]; **A** = [1450, 2140, 1360] MHz; hyperfine strain = [200, 0, 400] MHz; *g*_2_-strain = 0.2. [L(X)Ga]_2_Bi^•^: **g** = [2.61, 2.09, 1.73]; **A** = [1200, 2050, 900] MHz; *g*-strain = [0.03, 0.20, 0.01].

The X-band perpendicular-mode and Q-band EPR spectra of [L(I)Ga]_2_Bi^•^ were previously reported, however, we were unable to offer a complete interpretation of the experiment at the time.^11^ The breadth of the EPR spectra, along with magnetic susceptibility measurements, supported the *S* = 1/2 radical structure and localization of the electron to the bismuth center. As a result of long-term cryostorage at 77 K of the previously prepared X-band EPR sample, measurement of the parallel-mode X-band EPR spectrum was possible, Figure 3. This spectrum offers complementary information to the perpendicular-mode spectrum. Furthermore, the more recently isolated and characterized [L(Cl)Ga]_2_Bi^•^ radical was prepared for multifrequency and multimode EPR experiments, including a newly prepared W-band EPR sample.^14^ [L(Cl)Ga]_2_Bi^•^ and [L(I)Ga]_2_Bi^•^ exhibit similar 4 K X-band perpendicular- and parallel-mode EPR spectra (Figure S3) due to their similar electronic structures and the innocence of the gallium-coordinated halide. Additionally, the molecular Bi radical signals did not exhibit any microwave saturation behavior at 4 K. The innocence of these distant atoms to the central radical electronic structure was also previously demonstrated by EPR spectroscopy in the [L(X)Ga]_2_Sb^•^ (X = Cl, Br, and I) series.^31^ Because of the similar electronic structure of the bismuth radical centers in [L(Cl)Ga]_2_Bi^•^ and [L(I)Ga]_2_Bi^•^, these will simply be referred to as [L(X)Ga]_2_Bi^•^.

In addition to the neutral [L(X)Ga]_2_Bi^•^ radical, we will present here the EPR spectroscopic characterization of the heteroleptically coordinated bismuth radical cation [L(Cl)GaBi(^Me^cAAC)]^•+^. The X-band perpendicular-mode EPR spectra of [L(Cl)GaBi(^Me^cAAC)]^•+^ and [L(X)Ga]_2_Bi^•^ exhibit numerous broad transitions extending from 0 to above 7000 G, Figure 3. The X-band perpendicular-mode spectrum of [L(X)Ga]_2_Bi^•^ exhibits a superior signal-to-noise ratio, albeit broad features at the high field. The highest field feature at ∼6500 G places an upper limit for the evaluation of EPR parameters. A sharp signal is observed in each sample centered at *g* ∼ 2 that exhibits a different microwave saturation behavior (Figure S4), allowing us to putatively assign this to a minor paramagnetic impurity.

The X-band parallel-mode EPR spectra of the Bi radicals exhibit significant intensities and numerous transitions that are clearly different from those observed in the perpendicular-mode spectra. It is noted that the sharp signal at *g* ∼ 2 in the perpendicular-mode EPR is absent in the parallel-mode EPR spectrum, conclusively demonstrating that its origin is of another paramagnetic species. The same phenomenon is observed in the Si:Bi crystal (Figure 1) described above. The differences between the two microwave modes demonstrate the selection power of the technique to differentiate between *S* = 1/2 systems *with or without* very large hyperfine couplings.

The pulse detected Q-band (∼34 GHz) EPR spectrum of each Bi radical exhibits a broad EPR envelope (Figure S5), beginning at ∼7000 G and extending beyond the ∼14 000 G upper limit of the instrument’s magnet. The recorded absorption-like spectrum from the integration of the pulse echo is converted to the numerical derivative spectrum, Figure 3. For [L(Cl)GaBi(^Me^cAAC)]^•+^, at the low-field side of the Q-band EPR spectrum, hyperfine splitting from the Bi nuclei is well resolved, corresponding to a hyperfine coupling of *A*_1_(^209^Bi) = 1450 MHz. The spectrum also exhibits a sharp signal near *g* ∼ 2, the same impurity observed in the X-band perpendicular-mode EPR spectrum. The Q-band spectrum, while broad in the absorption, does not offer a distinct *g*_2_ turning point. Lastly, estimates of *g*_3_ and *A*_3_ are unattainable from the Q-band spectrum as they appear to extend well past the high-field limit of the instrument’s magnet. A well-resolved hyperfine pattern at the low-field edge of the Q-band spectrum is not observed for [L(X)Ga]_2_Bi^•^. Rather, the broad feature sets correlated limits for *g*_1_ and *A*_1_.

The higher-frequency echo-detected W-band (∼94.00 GHz) EPR spectrum (Figure S6) affords additional resolution of the *g*-values, and the larger 8 T magnet range of the instrument allows for the measurement of the *g*_3_ feature in each Bi radical species. The derivative of the echo-detected EPR spectra of [L(Cl)GaBi(^Me^cAAC)]^•+^ and [L(X)Ga]_2_Bi^•^ clearly exhibits a low-field feature with corresponding *g*_1_ values of 2.67 and 2.61, respectively, Figure 3. The *g*_1_ feature in each spectrum exhibits a square-like broadening due to the Bi hyperfine splitting. However, this hyperfine splitting is not as well resolved as seen in the Q-band spectrum, possibly due to microwave-induced strain.^32^ In agreement with the Q-band spectrum, the *g*_1_ feature of [L(Cl)GaBi(^Me^cAAC)]^•+^ is well simulated with a hyperfine coupling of *A*_1_(^209^Bi) = 1450 MHz. For [L(X)Ga]_2_Bi^•^, this coupling reduces to *A*_1_(^209^Bi) = 1200 MHz. At the high-field position of the W-band spectra, the *g*_3_ features are distinguished and exhibit broader line shapes than those observed for *g*_1_. [L(X)Ga]_2_Bi^•^ has a larger *g*_3_ value, 1.76, than [L(Cl)GaBi(^Me^cAAC)]^•+^, 1.56. Lastly, the W-band echo-detected EPR spectra offer no clear *g*_2_ position, similar to the Q-band echo-detected spectra. However, we do note that the sharper features centered near *g* = 2.05 and 2.00 are attributed to copper and manganese backgrounds from the W-band cavity, respectively (Figure S8). We speculate that possible unfavorable relaxation behavior of the radical such as extremely anisotropic relaxation may yield distorted intensities in the pulsed detected EPR spectra. In both the Q- and W-band measurements, phase memory times (measured along *g*_1_ and/or *g*_3_) were extremely short (<50 μs). Employment of longer repetition rates increased the relative intensity of responses that are characteristic for copper and manganese background signals (Figures S7, S8). Our attempts to measure continuous wave (CW) W-band EPR spectra were fruitless.

By simulation of the W-band spectrum, the positions of *g*_1_ and *g*_3_ and their respective widths due to hyperfine splitting are satisfactorily reproduced, Figure 3. Inclusion of *g*_2_ at the center of the EPR spectra with hyperfine splittings up to 2500 MHz does not influence the other turning points of the W-band spectrum. However, the Q-band spectrum sets an upper limit of the hyperfine coupling; otherwise, the low-field edge of the spectrum would appear to be at *even lower* magnetic field. These estimates from the higher frequency EPR spectra may be used with the remaining X-band perpendicular- and parallel-mode EPR data to further refine EPR parameters, particularly *g*_2_ and *A*_2_. Employing the EPR parameters resolved from the Q- and W-band experiments (*g*_1_, *g*_3_, *A*_1_, and *A*_3_), simulation of both the perpendicular- and parallel-mode EPR with values of *g*_2_ ∼ 1.95 and *A*_2_ = 2140 MHz for [L(Cl)GaBi(^Me^cAAC)]^•+^ and *g*_2_ = 2.09 and *A*_2_ = 2050 MHz for [L(X)Ga]_2_Bi^•^ yields spectra that match and align the experiment well. The X-band experimental and simulated spectra show the best agreement above ∼1000 G; however, slight discrepancies at the lowest field can be attributed to either possible baseline distortions or additional broadening that is not incorporated in the simulation.

**Figure 4:**
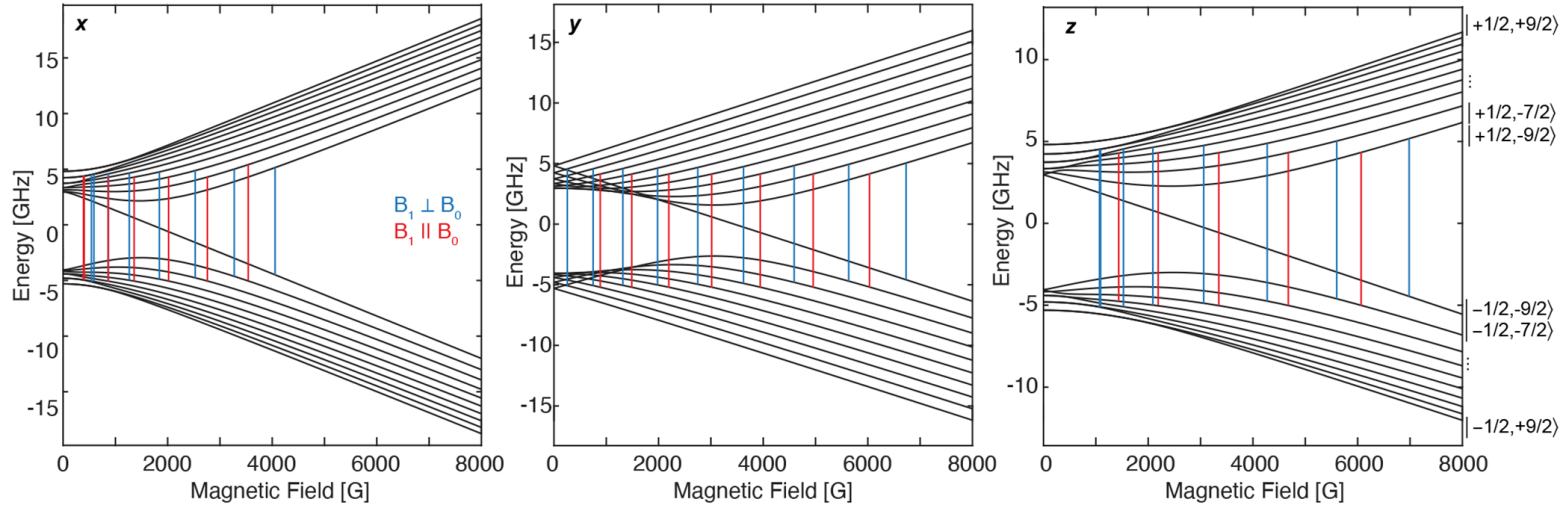
Energy level (Breit–Rabi) diagram of [L(Cl)GaBi(^Me^cAAC)]^•+^ along the conical molecular axes, *x*, *y*, and *z*, aligning with *g*_1_, *g*_2_, and *g*_3_, respectively. The predicted transitions are calculated from full **g** and **A**(Bi) tensors detailed in the caption of Figure 3. Both the perpendicular-mode (B_1_ ⊥ B_0_, ∼9.63 GHz) and parallel-mode (B_1_ ∥ B_0_, ∼9.34 GHz) EPR transitions are calculated at their experimental frequencies and marked by the vertical bars. Despite *M*_S_ and *M*_I_ not being good quantum numbers in the low-field experiment, the individual levels are labeled with the notation |*M*_S_, *M*_I_⟩ for ease of reading.

Similar relative patterns for the perpendicular- and parallel-mode transitions are observed in the Breit–Rabi energy diagrams along each of the molecular/conical *g* directions (Figure 4). The numerical calculation of the Breit–Rabi diagrams does, however, demonstrate the complexity of the Bi radical spectrum with both large **g** and **A** anisotropies. Here, a single unique solution to the X-band spectra would not be possible without the parameter restraints imposed from the higher frequency Q- and W-band measurements. In the range of 1000–3000 G of the X-band perpendicular- and parallel-mode EPR spectra, the sharpest features are observed. Additionally, these features exhibit the largest shifts in field relative to one another depending on the mode of the experiment. Inspection of the Breit–Rabi diagrams also shows the largest field position differences for individual transitions of the two microwave modes along the molecular *y* (*g*_2_) direction. This analysis further supports the fact that these shifting features observed in the experimental spectra are indeed the *A*_2_ hyperfine transitions not well-observed at higher microwave frequencies.

Besides, the previously characterized [O(SiMe_2_NAr)_2_]Bi^•^, [L(Cl)GaBi(^Me^cAAC)]^•+^, and [L(X)Ga]_2_Bi^•^ now represent the only stable mononuclear bismuth radicals characterized by EPR spectroscopy. It is apparent that the electronic structure of [O(SiMe_2_NAr)_2_]Bi^•^ is quite different from that of the gallium-coordinated Bi radicals studied here, Table 1. It is notable that for [O(SiMe_2_Nar)_2_]Bi^•^, each value of the *g*-tensor is less than *g*_e_, differing from that of both bismuth radicals studied here. The large *g*-anisotropy observed for [L(Cl)GaBi(^Me^cAAC)]^•+^ and [L(X)Ga]_2_Bi^•^ is not unlike that observed for the lighter Sb analogues; however, the low *g*_3_ value is unprecedented for a Bi radical and, more broadly, group 15 radicals.^12^ The hyperfine of [L(Cl)GaBi(^Me^cAAC)]^•+^ and [L(X)Ga]_2_Bi^•^ is significantly smaller than that observed for [O(SiMe_2_NAr)_2_]Bi^•^.^10^ It may be reasoned that the electropositive Ga ligands facilitate increased electron delocalization compared to the [O(SiMe_2_NAr)_2_] ligand, which coordinates *via* the lighter and more electronegative nitrogen atoms. Supported by previous density functional theory (DFT) calculations,^11−13^ the radicals [L(Cl)GaBi(^Me^cAAC)]^•+^ and [L(X)Ga]_2_Bi^•^ have been previously described as p-orbital-centered Bi radicals. Studies in analogous Ga-substituted Sb radicals evidence minimal isotropic spin density, as supported by small isotropic hyperfine couplings.^11,12,33^ The large isotropic hyperfine coupling constant of ^209^Bi, *a*_0_ ∼ 77,500 MHz,^34^ means that very minor differences in the isotropic spin density will profoundly modulate the isotropic hyperfine coupling. For [O(SiMe_2_NAr)_2_]Bi^•^, this estimates a Bi s-orbital spin density of ∼0.05. For [L(Cl)GaBi(^Me^cAAC)]^•+^ and [L(X)Ga]_2_Bi^•^, *a*_iso_ can be estimated in the range from +220 to +1650 MHz depending on the signs of the hyperfine components,^11,12^ corresponding to isotropic spin density estimates of ∼0 to 0.02. This vanishingly small isotropic spin density is consistent with the p-orbital-centered radical assignment. Although we do not make any absolute sign assignments for these Bi radicals, it is clear that the isotropic hyperfine couplings are comparably small and the s-orbital spin populations smaller than the ∼0.05 determined for [O(SiMe_2_NAr)_2_]Bi^•^.^10^ Attempts to estimate the p-orbital spin population of [L(Cl)GaBi(^Me^cAAC)]^•+^ and [L(X)Ga]_2_Bi^•^ result in maximum populations of ∼0.5, consistent with the interpretation of significant delocalization onto the ligand, and significantly smaller than the p-orbital population determined in the As and Sb analogues.^13^ A possible reason for this could be an enhanced spin delocalization onto the gallium ligand. Unfortunately, the EPR spectra do not offer sufficient resolution to determine the gallium hyperfine couplings and obtain estimates on the gallium spin populations.

**Table 1:** Summarized EPR Simulation Parameters for [O(SiMe_2_NAr)_2_]Bi^•10^, [L(Cl)GaBi(^Me^cAAC)]^•+^, and [L(X)Ga]_2_Bi^•^

	**g** = [*g*_1_*g*_2_*g*_3_]	**A**(^209^Bi) = [*A*_1_*A*_2_*A*_3_] (MHz)	
[O(SiMe_2_NAr)_2_]Bi^•^	[1.832, 1.676, 1.621]	[3830, 2804, 4764]	ref (10)
[L(Cl)GaBi(^Me^cAAC)]^•+^	[2.67, 1.95, 1.54]	[1450, 2140, 1360]	this work
[L(X)Ga]_2_Bi^•^	[2.61, 2.09, 1.73]	[1200, 2050, 900]	this work

**Figure 5:**
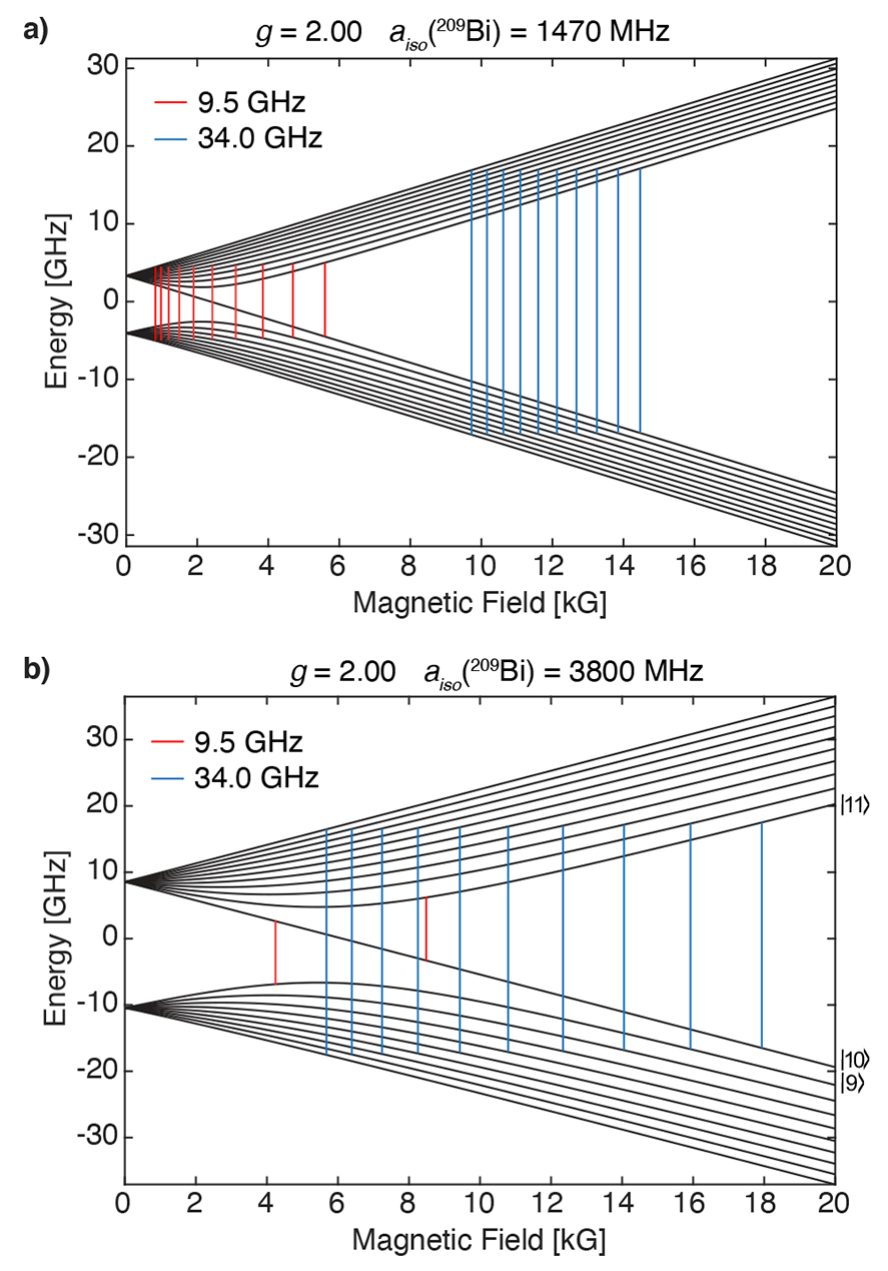
Breit–Rabi energy level diagrams with approximate multifrequency perpendicular-mode EPR transitions for (a) Si:Bi and (b) [O(SiMe_2_NAr)_2_]Bi^•^. An isotropic **g** and **A** are used for simple representation of the multifrequency differences anticipated for [O(SiMe_2_NAr)_2_]Bi^•^. The energy levels |9⟩, |10⟩, and |11⟩ are labeled in (b) without quantum numbers.

In terms of the experiment feasibility, the choice of microwave frequency is very important. We have already discussed that the parallel-mode EPR transition intensities will depend on the amount of state mixing. Also, the scale of the hyperfine interaction or the choice of the microwave energy does directly impact the ability to detect the numerous allowed transitions. Working at X-band frequency, we are able to observe all of the possible transitions for Si:Bi, which are at moderate fields and with inequivalent spacing of the transitions, Figure 5. At higher Q-band frequency, one begins to approach the high-field regime and retains all available transitions. This is the same case for [L(Cl)GaBi(^Me^cAAC)]^•+^ and [L(X)Ga]_2_Bi^•^ with similar hyperfine values. For [O(SiMe_2_Nar)_2_]Bi^•^, which has a much larger hyperfine coupling, all 10 transitions are observable at Q-band frequencies. However, at X-band frequencies, the number of allowed transitions decreases because the microwave energy is not sufficiently large. The only transitions in this low-field regime involve the uncoupled-state |10⟩, |9⟩ ↔ |10⟩ and |10⟩ ↔ |11⟩, Figure 5.^16^ These two transitions, labeled in Figure 5 for the case of *a*_iso_ = 3800 MHz at X-band frequencies, correspond to |9⟩ ↔ |10⟩ ≡ |−1/2, −7/2⟩ ↔ |−1/2, −9/2⟩ with only a flip of the nuclear spin and |10⟩ ↔ |11⟩ ≡ |−1/2, −9/2⟩ ↔ |+1/2, −9/2⟩ corresponding to only a flip of the electron spin. Both cases obey the Δ*M*_F_ = 1 selection rule, making them observable in perpendicular X-band EPR spectroscopy. This is why Schwamm et al. observed an X-band EPR spectrum of relatively few transitions but numerous transitions in the Q-band experiment.^10^ We do refer the interested reader to relevant qubit studies of Si:Bi and proposals of utilizing level |10⟩ as an initial state rather than the ground state.^16^ For EPR spectroscopic characterization, employing an EPR spectrometer with an increased microwave frequency in the moderate-field regime to better match the total angular momentum (**F** = **S** + **I**) energies will facilitate the observation of *all* possible allowed transitions in both perpendicular- and parallel-mode EPR spectroscopy.

## Conclusions

We have demonstrated the ability of parallel-mode EPR spectroscopy to offer complementary information to the more common perpendicular-mode EPR experiment for *S* = 1/2 systems of large hyperfine coupling. With this new approach, we report the full EPR characterization of [L(Cl)GaBi(^Me^cAAC)]^•+^ and [L(X)Ga]_2_Bi^•^ by conventional EPR laboratory techniques. While multifrequency EPR experiments serve to refine and offer excellent spectral information, the ability to collect dual-mode information on a single laboratory instrument for such complex systems is both attractive and advantageous to EPR spectroscopists. The commercial availability of dual-mode cavities and ease of the experiment presented here have an unmistakable appeal. High-frequency EPR spectroscopy, above that demonstrated here, still has excellent potential in the characterization of these bismuth radicals with large hyperfine couplings. Such high-frequency techniques have, with high resolution, characterized other *S* = 1/2 systems possessing an extreme hyperfine, such as the above-mentioned Lu(II) center with an isotropic hyperfine coupling of 3467 MHz.^30^ Coincidentally, the parallel-mode transitions of the Lu(II) center at the X-band frequency exhibit a 9.2 GHz clock transition with remarkably long relaxation rates. While the “pure” parallel-mode EPR spectrum of the lanthanide center was not reported, as dedicated parallel-mode pulsed EPR resonators are not common, our demonstration of the dramatic differences that can be observed between the two modes encourages both their implementation in CW EPR spectroscopy and future instrument development for pulsed parallel-mode EPR spectroscopy. For *S* = 1/2 centers with large hyperfine interactions compared to the g interaction, the ability to switch between perpendicular and parallel modes in pulsed EPR experiments is attractive for applications in quantum computing to better tune potential clock transitions of interest. In conclusion, the application of parallel-mode EPR to an *S* = 1/2 spin system is an unconventional utilization of the technique and warrants further considerations in other complex *S* = 1/2 systems. One may envision the development of a new or the modification of current low-frequency and broadband EPR spectrometers^35^ to perform parallel-mode EPR spectroscopy for *S* = 1/2 with large hyperfine couplings.

## Materials and Methods

### Bismuth-Doped Silicon

Single float-zone crystals of bismuth-doped silicon were made at the Leibniz-Institut für Kristallzüchtung (Berlin, Germany). The final crystal sizes were 2 × 2 × 4 mm and were made of naturally abundant Si with a Bi doping of 3.4 × 10^15^ cm^–3^. The crystals were loaded into the EPR spectrometer and held fixed to the bottom end of a quartz EPR tube by a minimal length of Teflon shrink tubing, covering approximately 1.5 mm of the top of the crystal.

### Molecular Bi Radicals

Previously prepared^11^ X-band EPR samples of [L(I)Ga]_2_Bi^•^ (10 mM in toluene) were recovered from long-term cryostorage (77 K) for new measurements. [L(Cl)GaBi(^Me^cAAC)]^•+^ and [L(Cl)Ga]_2_Bi^•^ were synthesized as previously described,^13,14^ and samples for EPR spectroscopy were prepared anaerobically as a 10 mM solution in fluorobenzene ([L(Cl)GaBi(^Me^cAAC)]^•+^) or a 5 mM solution in toluene ([L(Cl)Ga]_2_Bi^•^) and frozen in custom 0.9 mm (W-band), 2.8 mm (Q-band), and 4 mm (X-band) OD quartz EPR tubes.

### EPR Spectroscopy

CW X-band EPR spectra were measured on a Bruker E500 spectrometer equipped with an Oxford liquid helium flow cryostat. Spectra were collected in a dual-mode X-band resonator, operated in either the perpendicular-mode (TE_102_) or parallel-mode (TE_012_).

The CW X-band EPR spectra on the Si:Bi sample were collected at ∼25 K in perpendicular (∼9.65 GHz) and parallel (∼9.37 GHz) modes with 100 kHz and 1 G field modulation and the following parameters: full width spectra: time constant = 20.48 ms, sweep time = 671 s, number of points = 8192, number of scans = 4 and 12 for perpendicular- and parallel-modes, respectively. Spectra of each individual transition: time constant = 81.92 ms, sweep time = 168 s, number of points = 512, number of scans = 2 and 5 to 13 for perpendicular- and parallel-modes, respectively.

CW X-band EPR spectra of the molecular Bi radicals were measured at ∼4 K in perpendicular- (∼9.63 GHz) and parallel-modes {∼9.33 GHz ([L(X)Ga]_2_Bi^•^), ∼9.34 GHz ([L(Cl)GaBi(^Me^cAAC)]^•+^)} with the following parameters: field modulation frequency = 100 kHz, field modulation amplitude = 6 G, time constant = 81.92 ms, sweep time = 336 s, number of points = 4096. For spectra in the perpendicular-mode, one ([L(X)Ga]_2_Bi^•^) and six scans ([L(Cl)GaBi(^Me^cAAC)]^•+^) were collected, respectively. For spectra in the parallel-mode, 10 ([L(Cl)Ga]_2_Bi^•^), 4 ([L(I)Ga]_2_Bi^•^), and 20 scans ([L(Cl)GaBi(^Me^cAAC)]^•+^) were collected. The spectra of [L(Cl)GaBi(^Me^cAAC)]^•+^ at several microwave powers (Figure S4) were obtained under the same conditions but with 1024 points and sweep times of 168 s, respectively.

Q-band {∼33.98 GHz ([L(Cl)GaBi(^Me^cAAC)]^•+^), ∼34.02 GHz ([L(Cl)Ga]_2_Bi^•^)} pulsed EPR spectra were collected on a Bruker Elexsys E580 spectrometer equipped with a home-built up/down Q-band pulse conversion accessory,^36^ a cylindrical TE_011_ microwave resonator,^37^ and an Oxford CF935 helium flow cryostat and temperature controller. The spectra were obtained with a two-pulse Hahn sequence (π/2−τ–π–τ–echo) with the following parameters: For [L(Cl)GaBi(^Me^cAAC)]^•+^: temperature = 6 K, π = 80 ns, repetition rate = 300 us, shots per point = 25, number of points = 4096, τ was varied between 300 and 650 ns, and the respective spectra summed with three scans for each value. For [L(Cl)Ga]_2_Bi^•^: temperature = 7 K, π = 32 ns, repetition rate = 250 us, shots per point = 250, number of points = 8192, τ was varied between 300 and 600 ns, and the respective spectra summed with one scan for each value.

W-band {∼94.00 GHz ([L(Cl)GaBi(^Me^cAAC)]^•+^), ∼94.04 GHz ([L(Cl)Ga]_2_Bi^•^)} pulsed EPR measurements were collected on a Bruker Elexsys E680 spectrometer with a closed cycle helium cryostat system at 6 K ([L(Cl)GaBi(^Me^cAAC)]^•+^) and 10 K ([L(Cl)Ga]_2_Bi^•^), respectively. The spectra were collected with a two-pulse Hahn sequence (π/2−τ–π–τ–echo), applying the following parameters: π = 40 ns, repetition rate = 500 us, shots per point = 1024, number of points = 5200, τ was varied between 300 and 600 ns, and the respective spectra were summed. The magnet was swept up and down at the same sweep rate for each τ value, and the offsets were averaged to account for sweep delays.

All energy level diagrams, transitions, and EPR simulations were performed in Matlab with the EasySpin (v 6.0.0) package.^38^

## Supporting Information Available

Breit–Rabi energy diagram of the ^1^H atom at 2.0 and 9.5 GHz; Si:Bi frequency-dependent EPR intensity calculations; comparisons of X-band EPR spectra of [L(Cl)Ga]_2_Bi^•^ and [L(I)Ga]_2_Bi^•^; and supplemental Q- and W-band pulsed EPR experiments (PDF)

## References

[ref1] Lichtenberg C. Molecular bismuth(III) monocations: structure, bonding, reactivity, and catalysis. Chem. Commun. 2021, 57, 4483–4495. 10.1039/d1cc01284c. 33861277

[ref2] Lichtenberg C. Well-Defined, Mononuclear Bi^I^ and Bi^II^ Compounds: Towards Transition-Metal-Like Behavior. Angew. Chem., Int. Ed. 2016, 55, 484–486. 10.1002/anie.201509234. 26630322

[ref3] Helling C.; Schulz S. Long-Lived Radicals of the Heavier Group 15 Elements Arsenic, Antimony, and Bismuth. Eur. J. Inorg. Chem. 2020, 2020, 3209–3221. 10.1002/ejic.202000571.

[ref4] Lichtenberg C. Radical Compounds of Antimony and Bismuth. In Encyclopedia of Inorganic and Bioinorganic Chemistry; Wiley, 2020; pp 1–12.

[ref5] Oberdorf K.; Hanft A.; Ramler J.; Krummenacher I.; Bickelhaupt F. M.; Poater J.; Lichtenberg C. Bismuth Amides Mediate Facile and Highly Selective Pn-Pn Radical-Coupling Reactions (Pn = N, P, As). Angew. Chem., Int. Ed. 2021, 60, 6441–6445. 10.1002/anie.202015514. PMC7986226 33315293

[ref6] Schwamm R. J.; Lein M.; Coles M. P.; Fitchett C. M. Catalytic oxidative coupling promoted by bismuth TEMPOxide complexes. Chem. Commun. 2018, 54, 916–919. 10.1039/c7cc08402a. 29318242

[ref7] Ishida S.; Hirakawa F.; Furukawa K.; Yoza K.; Iwamoto T. Persistent Antimony- and Bismuth-Centered Radicals in Solution. Angew. Chem., Int. Ed. 2014, 53, 11172–11176. 10.1002/anie.201405509. 25066471

[ref8] Ishida S.; Hirakawa F.; Iwamoto T. A Series of Two-Coordinate Group-15 Element (P, As, Sb, Bi) Centered Radicals Having Bulky Alkyl Groups. Bull. Chem. Soc. Jpn. 2018, 91, 1168–1175. 10.1246/bcsj.20180057.

[ref9] Weinert H. M.; Wölper C.; Haak J.; Cutsail G. E. III; Schulz S. Synthesis, structure and bonding nature of heavy dipnictene radical anions. Chem. Sci. 2021, 12, 14024–14032. 10.1039/d1sc04230k. 34760185 PMC8565390

[ref10] Schwamm R. J.; Harmer J. R.; Lein M.; Fitchett C. M.; Granville S.; Coles M. P. Isolation and Characterization of a Bismuth(II) Radical. Angew. Chem., Int. Ed. 2015, 54, 10630–10633. 10.1002/anie.201504632. 26215838

[ref11] Ganesamoorthy C.; Helling C.; Wölper C.; Frank W.; Bill E.; Cutsail G. E. III; Schulz S. From stable Sb- and Bi-centered radicals to a compound with a Ga=Sb double bond. Nat. Commun. 2018, 9, 87 10.1038/s41467-017-02581-2. 29311607 PMC5758792

[ref12] Cutsail G. E. III. Applications of electron paramagnetic resonance spectroscopy to heavy main-group radicals. Dalton Trans. 2020, 49, 12128–12135. 10.1039/d0dt02436h. 32812583

[ref13] Krüger J.; Haak J.; Wölper C.; Cutsail G. E. III; Haberhauer G.; Schulz S. Single-Electron Oxidation of Carbene-Coordinated Pnictinidenes-Entry into Heteroleptic Radical Cations and Metalloid Clusters. Inorg. Chem. 2022, 61, 5878–5884. 10.1021/acs.inorgchem.2c00249. 35333051

[ref14] Krüger J.; Wölper C.; Schulz S. Stepwise Bi-Bi Bond Formation: From a Bi-centered Radical to Bi4 Butterfly and Bi8 Cuneane-Type Clusters. Inorg. Chem. 2020, 59, 11142–11151. 10.1021/acs.inorgchem.0c01657. 32663023

[ref15] Feher G. Electron Spin Resonance Experiments on Donors in Silicon. I. Electronic Structure of Donors by the Electron Nuclear Double Resonance Technique. Phys. Rev. 1959, 114, 1219–1244. 10.1103/physrev.114.1219.

[ref16] Mohammady M. H.; Morley G. W.; Monteiro T. S. Bismuth Qubits in Silicon: The Role of EPR Cancellation Resonances. Phys. Rev. Lett. 2010, 105, 067601–067604. 10.1103/physrevlett.105.067602. 20868015

[ref17] Pica G.; Wolfowicz G.; Urdampilleta M.; Thewalt M. L. W.; Riemann H.; Abrosimov N. V.; Becker P.; Pohl H.-J.; Morton J. J. L.; Bhatt R. N.; Lyon S. A.; Lovett B. W. Hyperfine Stark effect of shallow donors in silicon. Phys. Rev. B 2014, 90, 195204 10.1103/physrevb.90.195204. 25375741

[ref18] Mortemousque P. A.; Berger S.; Sekiguchi T.; Culan C.; Elliman R. G.; Itoh K. M. Hyperfine clock transitions of bismuth donors in silicon detected by spin-dependent recombination. Phys. Rev. B 2014, 89, 161 10.1103/physrevb.89.155202.

[ref19] Mohammady M. H.; Morley G. W.; Nazir A.; Monteiro T. S. Analysis of quantum coherence in bismuth-doped silicon: A system of strongly coupled spin qubits. Phys. Rev. B 2012, 85, 094404–094416. 10.1103/physrevb.85.094404.

[ref20] Morley G. W.; Warner M.; Stoneham A. M.; Greenland P. T.; van Tol J.; Kay C. W. M.; Aeppli G. The initialization and manipulation of quantum information stored in silicon by bismuth dopants. Nat. Mater. 2010, 9, 725–729. 10.1038/nmat2828. 20711180

[ref21] Petasis D. T.; Hendrich M. P. A new Q-band EPR probe for quantitative studies of even electron metalloproteins. J. Magn. Reson. 1999, 136, 200–206. 10.1006/jmre.1998.1657. 9986761

[ref22] Hendrich M. P.; Debrunner P. G. EPR spectra of quintet ferrous myoglobin and a model heme compound. J. Magn. Reson. 1988, 78, 133–141. 10.1016/0022-2364(88)90163-1.

[ref23] Hendrich M. P.; Debrunner P. G. Integer-spin electron paramagnetic resonance of iron proteins. Biophys. J. 1989, 56, 489–506. 10.1016/S0006-3495(89)82696-7. 2551404 PMC1280502

[ref24] Pierce B. S.; Elgren T. E.; Hendrich M. P. Mechanistic implications for the formation of the diiron cluster in ribonucleotide reductase provided by quantitative EPR spectroscopy. J. Am. Chem. Soc. 2003, 125, 8748–8759. 10.1021/ja021290h. 12862469

[ref25] Marts A. R.; Greer S. M.; Whitehead D. R.; Woodruff T. M.; Breece R. M.; Shim S. W.; Oseback S. N.; Papish E. T.; Jacobsen F. E.; Cohen S. M.; Tierney D. L. Dual Mode EPR Studies of a Kramers ion: High-Spin Co(II) in 4-, 5- and 6-Coordination. Appl. Magn. Reson. 2011, 40, 501–511. 10.1007/s00723-011-0225-5.

[ref26] Piligkos S.; Collison D.; Oganesyan V. S.; Rajaraman G.; Timco G. A.; Thomson A. J.; Winpenny R. E. P.; McInnes E. J. L. Single-crystal parallel-mode EPR spectroscopy of an *S* = 6 ground-state transition-metal cluster. Phys. Rev. B 2004, 69, 134424 10.1103/physrevb.69.134424.

[ref27] Weil J. A. The hydrogen atom, revisited: Parallel-field magnetic resonance. Concepts Magn. Reson., Part A 2006, 28A, 331–336. 10.1002/cmr.a.20062.

[ref28] Weil J. A.; Bolton J. R., Electron Paramagnetic Resonance: Elementary Theory and Practical Applications, 2nd ed.; Wiley-Interscience: Hoboken, NJ, 2007.

[ref29] Mitrikas G.; Sanakis Y.; Ioannidis N. Parallel-Mode EPR of Atomic Hydrogen Encapsulated in POSS Cages. Appl. Magn. Reson. 2020, 51, 1451–1466. 10.1007/s00723-020-01263-5.

[ref30] Kundu K.; White J. R. K.; Moehring S. A.; Yu J. M.; Ziller J. W.; Furche F.; Evans W. J.; Hill S. A 9.2-GHz clock transition in a Lu(II) molecular spin qubit arising from a 3,467-MHz hyperfine interaction. Nat. Chem. 2022, 14, 392–397. 10.1038/s41557-022-00894-4. 35288686

[ref31] Helling C.; Wölper C.; Cutsail G. E. III; Haberhauer G.; Schulz S. A Mechanistic Study on Reactions of Group 13 Diyls LM with Cp*SbX 2 : From Stibanyl Radicals to Antimony Hydrides. Chem.—Eur. J. 2020, 26, 13390–13399. 10.1002/chem.202001739. 32428370 PMC7693246

[ref32] Hagen W. R.; Albracht S. P. J. Analysis of Strain-Induced EPR-Line Shapes and Anisotropic Spin-Lattice Relaxation in a [2Fe-2S] Ferredoxin. Biochim. Biophys. Acta 1982, 702, 61–71. 10.1016/0167-4838(82)90027-9. 6279164

[ref33] Helling C.; Cutsail G. E. III; Weinert H.; Wölper C.; Schulz S. Ligand Effects on the Electronic Structure of Heteroleptic Antimony-Centered Radicals. Angew. Chem., Int. Ed. 2020, 59, 7561–7568. 10.1002/anie.202000586. PMC7216903 32048388

[ref34] Morton J. R.; Preston K. F. Atomic parameters for paramagnetic resonance data. J. Magn. Reson. 1978, 30, 577–582. 10.1016/0022-2364(78)90284-6.

[ref35] Hagen W. R. Very Low-Frequency Broadband Electron Paramagnetic Resonance Spectroscopy of Metalloproteins. J. Phys. Chem. C 2021, 125, 3208–3218. 10.1021/acs.jpca.1c01217. PMC8154605 33848159

[ref36] Judd M.; Jolley G.; Suter D.; Cox N.; Savitsky A. Dielectric Coupler for General Purpose Q-Band EPR Cavity. Appl. Magn. Reson. 2021, 10.1007/s00723-021-01404-4.

[ref37] Reijerse E.; Lendzian F.; Isaacson R.; Lubitz W. A tunable general purpose Q-band resonator for CW and pulse EPR/ENDOR experiments with large sample access and optical excitation. J. Magn. Reson. 2012, 214, 237–243. 10.1016/j.jmr.2011.11.011. 22196894

[ref38] Stoll S.; Schweiger A. EasySpin, a comprehensive software package for spectral simulation and analysis in EPR. J. Magn. Reson. 2006, 178, 42–55. 10.1016/j.jmr.2005.08.013. 16188474

