# Supplementary material for: X-Band Parallel-Mode and Multifrequency Electron Paramagnetic Resonance Spectroscopy of S = 1/2 Bismuth Centers
Source: Inorg Chem. 2022 Jul 14;61(29):11173–81. doi: 10.1021/acs.inorgchem.2c01141 (PMC9326968; doi:10.1021/acs.inorgchem.2c01141)
Supplement: Supplementary file 1 — ic2c01141_si_001.pdf [file ic2c01141_si_001.pdf]

Supplemental Information

for

X-Band Parallel-Mode and Multifrequency Electron Paramagnetic Resonance Spectroscopy of *S* = 1/2 Bismuth Centers

Julia Haak,[1,2] Julia Krüger,[2,3] Nikolay V. Abrosimov,[4] Christoph Helling,[2,3] Stephan Schulz,[2,3] George E. Cutsail III[1,2*]

[1] Max Planck Institute for Chemical Energy Conversion (CEC), Stiftstraße 34–36, 45470 Mülheim an der Ruhr (Germany)

[2] Institute of Inorganic Chemistry and [3]Center for Nanointegration Duisburg-Essen (CENIDE), University of Duisburg-Essen, Universitätsstraße 5-7, 45141 Essen (Germany)

[4] Leibniz-Institut für Kristallzüchtung, Max-Born Strasse 2, 12489 Berlin (Germany)

*email: george.cutsail@cec.mpg.de

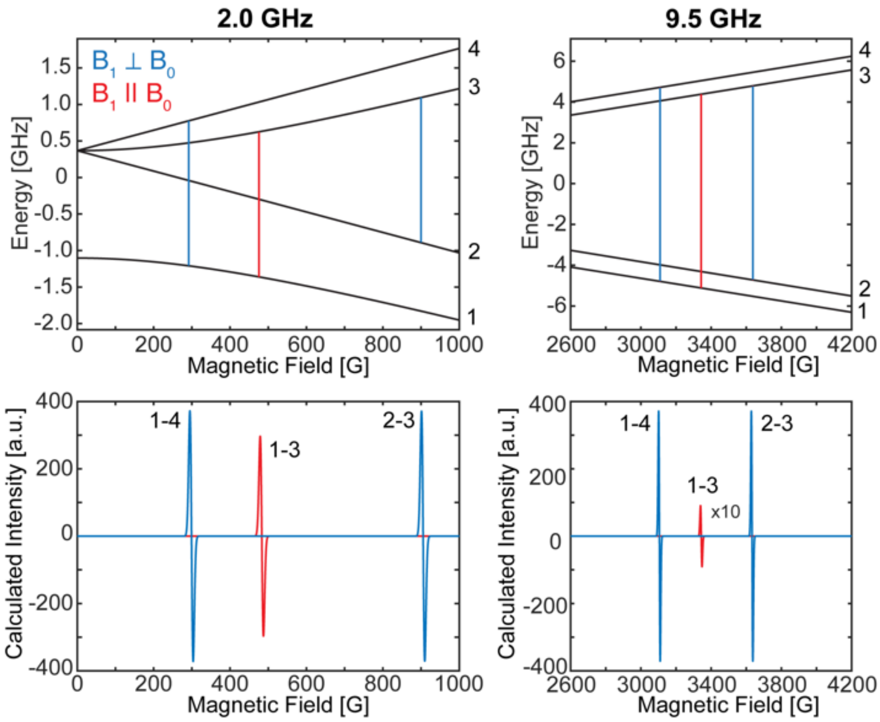

Figure S1. Breit-Rabi energy diagrams (top) of the ^{1}H atom and allowed transitions in both perpendicular- (blue) and parallel-mode (red) EPR spectroscopy. The diagrams are shown at both the low-field regime (left) with transitions corresponding to 2.0 GHz microwave incident energy, and intermediate/high-field at 9.5 GHz. The calculated EPR spectra are shown (bottom) for each EPR detection mode at both 2.0 and 9.5 GHz. The absolute calculated EPR intensities are displayed. The simulated lines are broadened by 10 G (full-width half-max) for ease of inspection.

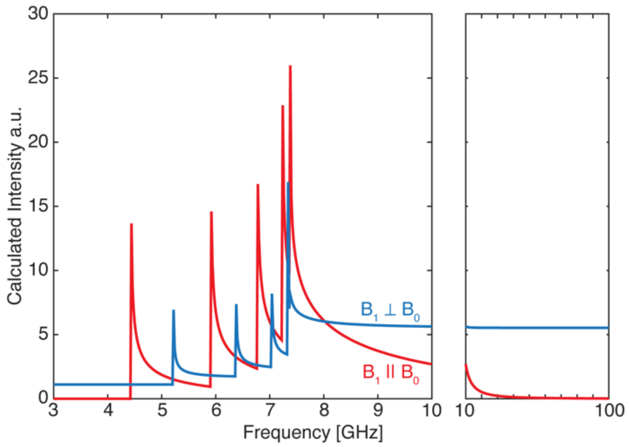

Figure S2. EPR intensity vs. frequency plot for the Si:Bi system.

This EPR intensity vs. frequency plot has clear discontinuities related to the number of allowed transitions. For instance, immediately below ~4.3 GHz, the perpendicular-mode EPR has a given intensity from the two allowed transitions, whereas there are no allowed transitions in the parallel-mode EPR calculation. At ~4.3 GHz, one observes an instant rise of intensity for the parallel-mode EPR calculations. Subsequently, the intensity decreases with increasing frequency until 5.9 GHz, where an additional two transitions become allowed in the calculation (similar to Figure 5b). This pattern continues until all nine allowed transitions in the parallel-mode calculation are at their maximum intensity at 7.6 GHz. A similar toothed-pattern is observed for the perpendicular-mode EPR calculation, also achieving a maximum intensity around 7.6 GHz. This diagram suggests that dual-mode collection of the Si:Bi sample at a microwave frequency of ~7.6 GHz would maximize the intensity of each collected spectrum.

The calculated intensity of the EPR spectrum as a function of frequency also clearly demonstrates two differing behaviours for the perpendicular- and parallel-mode calculations as one moves to the high-field regime (higher frequency). The perpendicular-mode EPR response intensity is expected to plateau and remain constant, as demonstrated by the linear intensity response above 10 GHz. However, the intensity of the parallel-mode EPR response continues to decrease as the degree of state-mixing (electron and nuclear spin-state mixing) diminishes as one moves to higher frequency (and higher magnetic fields).

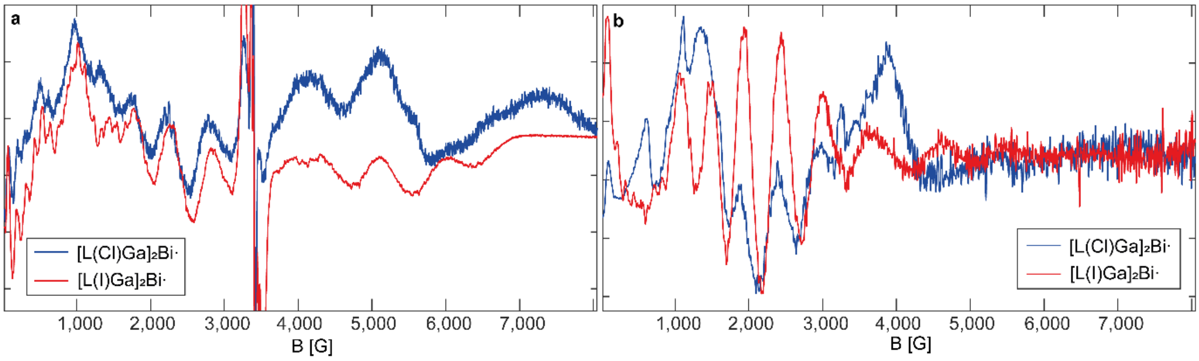

Figure S3. CW X-band (~9.63 GHz) EPR spectra of $[L(Cl)Ga]_2Bi^{\bullet}$ and $[L(I)Ga]_2Bi^{\bullet}$ in perpendicular- (**a**) and parallel- (**b**) mode, showing very similar EPR responses due to the negligible influence of the distant halide on the electronic structure of the radicals. Spectra in parallel-mode were smoothed over 15 ($[L(Cl)Ga]_2Bi^{\bullet}$) and 10 ($[L(I)Ga]_2Bi^{\bullet}$) points, respectively. Baseline distortions in $[L(Cl)Ga]_2Bi^{\bullet}$ are partially attributed to background signal which appears more pronounced in less concentrated samples.

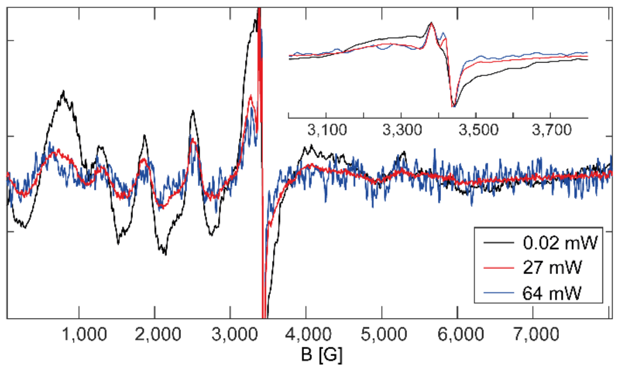

Figure S4. CW X-band (~9.63 GHz) EPR spectra of [L(Cl)GaBi(^{Me}cAAC)]$^{\bullet+}$ in perpendicular-mode at several microwave powers, scaled to the intensity of the sharp signal at g≈2. The spectra show saturation and therefore suppression of the Bi transitions at higher microwave power compared to the sharp signal, which can consequently be attributed to a minor paramagnetic impurity. The spectra are smoothed over 1 point.

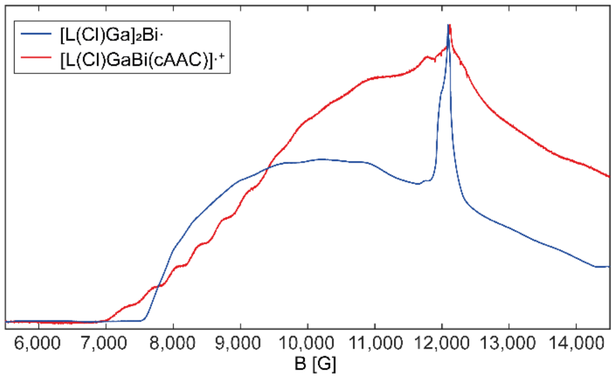

Figure S5. As collected pulsed Q-band EPR absorption spectra of [L(Cl)GaBi(^{Me}cAAC)]$^{•+}$ (~33.98 GHz) and [L(Cl)Ga]$_2$Bi$^•$ (~34.02 GHz).

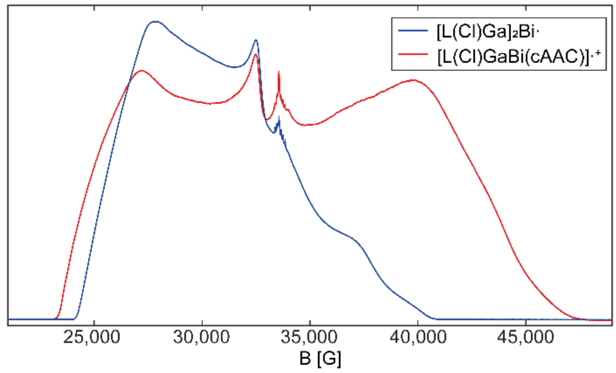

Figure S6. As collected pulsed W-band EPR absorption spectra of [L(Cl)GaBi(^{Me}cAAC)]$^{•+}$ (~94.00 GHz) and [L(Cl)Ga]$_2$Bi$^{•}$ (~94.04 GHz).

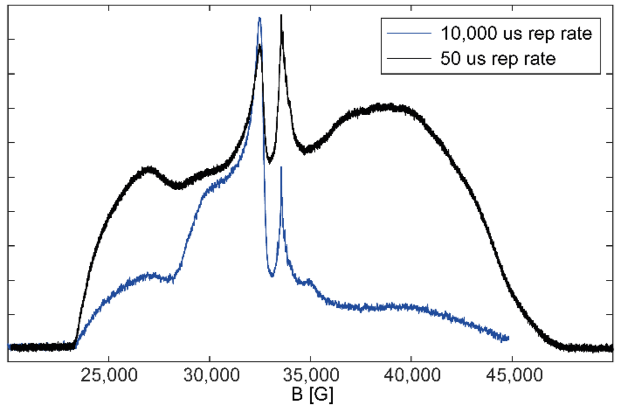

Figure S7. As collected pulsed W-band (~94.00 GHz) spectra of [L(Cl)GaBi(^{Me}cAAC)]$^{•+}$, obtained with repetition rates of 50 us and 10,000 us. Longer repetition rates increase the relative intensity of the copper and manganese background (see Figure S8).

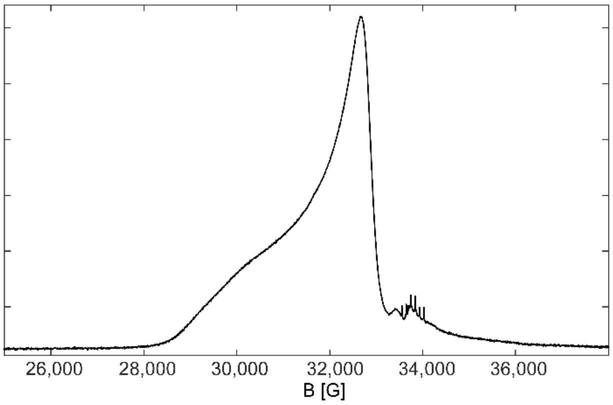

Figure S8. As collected pulsed W-band (~94.00 GHz) spectrum of the resonator background showing copper (~28,000 G – 33,300 G) and manganese (33,500 G – 34,100 G) impurities.
